# Integrated Array Tomography for 3D Correlative Light and Electron Microscopy

**DOI:** 10.3389/fmolb.2021.822232

**Published:** 2022-01-19

**Authors:** Ryan Lane, Anouk H. G. Wolters, Ben N. G. Giepmans, Jacob P. Hoogenboom

**Affiliations:** ^1^ Imaging Physics, Delft University of Technology, Delft, Netherlands; ^2^ Department of Biomedical Sciences of Cells and Systems, University Groningen, University Medical Center Groningen, Groningen, Netherlands

**Keywords:** correlative light and electron microscopy, volume electron microscopy, integrated microscopy, array tomography, serial section electron microscopy, scanning electron microscopy

## Abstract

Volume electron microscopy (EM) of biological systems has grown exponentially in recent years due to innovative large-scale imaging approaches. As a standalone imaging method, however, large-scale EM typically has two major limitations: slow rates of acquisition and the difficulty to provide targeted biological information. We developed a 3D image acquisition and reconstruction pipeline that overcomes both of these limitations by using a widefield fluorescence microscope integrated inside of a scanning electron microscope. The workflow consists of acquiring large field of view fluorescence microscopy (FM) images, which guide to regions of interest for successive EM (integrated correlative light and electron microscopy). High precision EM-FM overlay is achieved using cathodoluminescent markers. We conduct a proof-of-concept of our integrated workflow on immunolabelled serial sections of tissues. Acquisitions are limited to regions containing biological targets, expediting total acquisition times and reducing the burden of excess data by tens or hundreds of GBs.

## Introduction

A central objective within neuroscience and cell biology is to produce high-resolution (1–10 nm), three-dimensional reconstructions of biological specimen. Volume electron microscopy (EM) is the preferred imaging method in this arena because of its unique ability to resolve features across a wide spectrum of spatial scales (Peddie and Collinson, 2014; Kornfeld and Denk, 2018). While EM provides highly relevant structural information and precise localization of targets, immunogold labeling can only be visualized at high resolution, and in Tokuyasu labeling section areas are typically limited to 0.01 mm^2^ for analysis (Liou et al., 1996; Van Rijnsoever et al., 2008). Fluorescence microscopy (FM) provides biologically relevant information by tagging specific biomolecules with fluorescent labels at large scale (Giepmans et al., 2006). Regions of interest (ROI) can in this way be quickly and reliably identified for subsequent high magnification EM imaging. The information from these two imaging modalities are combined in correlative light and electron microscopy (CLEM). ROI retrieval across different microscopes is, however, nontrivial at large scales, particularly when spread across multiple sections (Polishchuk et al., 2000; Bishop et al., 2011; Karreman et al., 2014; Collinson et al., 2017; Booth et al., 2019). Other challenges associated with CLEM include the reliance on fiducial markers and intermediate sample preparation (de Boer et al., 2015; Karreman et al., 2016). One means of combating these challenges is by merging these separate imaging systems into a single, integrated fluorescence and electron microscope (Liv et al., 2013). By detecting fluorescence expression *in-situ*, it can further be decided in an automated fashion which areas to scan at high magnification and which areas to omit for the sake of higher throughput (Delpiano et al., 2018). For array tomography applications, ROIs can be targeted with increasing magnification through a sequence of feedback loops (Gabarre et al., 2021). Similarly, strategies for rapidly screening sections have been developed for sequential CLEM to limit volume acquisitions to select ROI (Burel et al., 2018; Ronchi et al., 2021).

Despite these potential benefits, an integrated microscope presents new challenges. In conventional array tomography sample preparation, the sample is eluted and restained between imaging methods (Micheva and Smith, 2007). Hence, there is no need to preserve fluorescence labelling, which allows for post-staining to enhance EM contrast (Watson, 1958; Tapia et al., 2012). The traditional way to compensate for diminished contrast is to boost the EM signal by increasing the dwell time per pixel, but this comes at the expense of throughput. An additional complication in integrated CLEM is electron-beam-induced quenching of the fluorescence (Srinivasa Raja et al., 2021). This imposes the constraint that the fluorescence in a given area must be acquired prior to exposure from the electron beam, which prohibits uniformly pre-irradiating the sample with the electron beam to enhance and stabilize contrast (Kuipers et al., 2015). Conversely, in conventional serial-section EM, there are scarce constraints regarding the number of times a particular sample may be scanned, making possible approaches such as that by Hildebrand et al. (Hildebrand et al., 2017).

Our goal is to establish a workflow capable of quickly and efficiently rendering three-dimensional CLEM volumes from serial sections in such a way as to overcome these challenges. Three key initiatives steered the design of our integrated correlative array tomography (iCAT) procedure. First, to prevent damaging or quenching of the fluorescence signal via electron-beam irradiation, each FM field of view must be acquired prior to EM exposure. Second, to compensate for the reduced application of contrast agents, backscattered electron (BSE) collection efficiency is enhanced via a negative stage bias, allowing for higher throughput (Bouwer et al., 2016; Lane et al., 2021). Finally, a high precision EM-FM overlay is facilitated by the use of cathodoluminescent (CL) points, which eliminates the need for artificial fiducial markers (Haring et al., 2017). An alignment method was then developed to reconstruct the correlative image stack. Islets of Langerhans from both rat and zebrafish pancreas tissue were chosen to prototype the imaging and reconstruction workflows. By offering a more holistic visualization of tissue, our integrated approach to 3D CLEM could lead to greater insights in (patho)biology (de Boer et al., 2020).

## Materials and Methods

### Tissue and Sample Preparation

Rat pancreas was prepared as follows: fresh pancreas was cut from an 83 day old rat into small pieces and fixed in 4% paraformaldehyde (PFA; Merck) + 0.1% glutaraldehyde (GA; Polysciences) as described in Ravelli et al. (Ravelli et al., 2013). A complete zebrafish larva (120hpf) was fixed in 2% PFA +2% GA. Both samples were post-fixed in 1% osmium tetroxide and 1.5% potassium ferricyanide in 0.1 M cacodylate buffer, dehydrated through ethanol and embedded in EPON (Serva). 100nm serial sections were cut and placed onto formvar-covered ITO-coated glass coverslips (Optics Balzers). Immunolabeling was performed as described previously (Kuipers et al., 2015). Samples were etched with 1% periodic acid for 10 min, followed by a 30 min blocking step: 1% bovine serum albumin (BSA; Sanquin, Netherlands) in tris-buffered saline (TBS), pH 7.4. Next, anti-insulin was incubated for 2 h (guinea pig; 1:50, Invitrogen, PA1-26938, RRID: AB_794668, for rat pancreas and anti-insulin; 1:100, Abcam, ab210560, for zebrafish pancreas), followed by washing and subsequent incubation for 1 h with biotinylated secondary antibody (donkey-anti-guinea pig; 1:400, Jackson Immunoresearch, for rat pancreas and goat-anti-rabbit; 1:400, Dako, for zebrafish pancreas) followed by washing steps. Finally, streptavidin conjugated AF594 (1:100, Jackson Immunoresearch, for rat pancreas) and streptavidin conjugated TRITC (1:100, Jackson Immunoresearch, for zebrafish pancreas) were added for 1 h followed by washing.

### Digital Light Microscopy

The sections, after being placed on the ITO-coated glass slide, are imaged at 30X magnification (∼7 μm px^−1^) using a VHX-6000 digital light microscope (Keyence) operating in reflection mode. To capture every section on the 22 mm × 22 mm ITO-coated glass slide, a 3 × 3 grid of RGB images is acquired and automatically stitched together.

### Integrated Microscopy

The integrated microscope is a widefield SECOM fluorescence microscope (Delmic B.V.) retrofitted into the vacuum chamber of a Verios 460 SEM (Thermo Fisher Scientific) (Liv et al., 2013; Zonnevylle et al., 2013). The microscopes share a common optical axis, translation stage, and control software. FM images are obtained with 10s exposures, recorded by a Zyla 4.2 sCMOS camera (Andor—Oxford Instruments). Excitation wavelengths of 405 and 555 nm are used to excite Hoechst and AF594. The SECOM is equipped with a CFI S Plan Fluor ELWD 60XC (0.70 NA) objective (Nikon), which allows for long working distance imaging (1.8–2.6 mm), to prevent electrical breakdown in vacuum, which must be accounted for due to the presence of high electric fields induced by the stage bias (Vos et al., 2021).

SEM imaging is conducted in two rounds: 1) low-magnification (38 nm px^−1^) scans accompanying each fluorescent acquisition; 2) high-magnification (5 nm px^−1^) acquisitions on ROI identified by fluorescence expression. Both low and high magnification imaging are performed at 2.5 keV primary beam energy with a −1 kV bias potential applied to the sample stage such that the landing energy is 1.5 keV, which proved optimal for ∼100 nm sections. The negative potential bias enhances the backscattered electron (BSE) signal, which is collected by the insertable concentric backscattered detector (Thermo Fisher Scientific) (Lane et al., 2021).

### Alignment and Reconstruction Software

Image data from the integrated microscope is uploaded to a local storage server running an instance of render-ws^1^, a collection of open-source web services for rendering transformed image tiles. The tiles and their respective metadata are organized into stacks, configured as MongoDB databases. The alignment routines are arranged in a series of Jupyter notebooks^2^, which parse the image metadata for the EM-FM overlay as well as make calls to render-ws via a python wrapper (render-python^3^). EM image stitching and volume alignment are based on the scale-invariant feature transform (SIFT)—an algorithm designed to detect and match local features in corresponding images (Lowe, 1999). SIFT features are stored in render-ws databases where they can be processed by BigFeta^4^, a linear least squares solver for scalable 2D and 3D image alignment based on point correspondences. CLEM datasets are ultimately exported to CATMAID (Saalfeld et al., 2009) for google-maps-like visualization. 3D visualizations are done in Fiji (Schindelin et al., 2012) using the Volume Viewer plugin^5^.

## Results

### Section Detection and *In-Situ* Navigation

**FIGURE 1:**
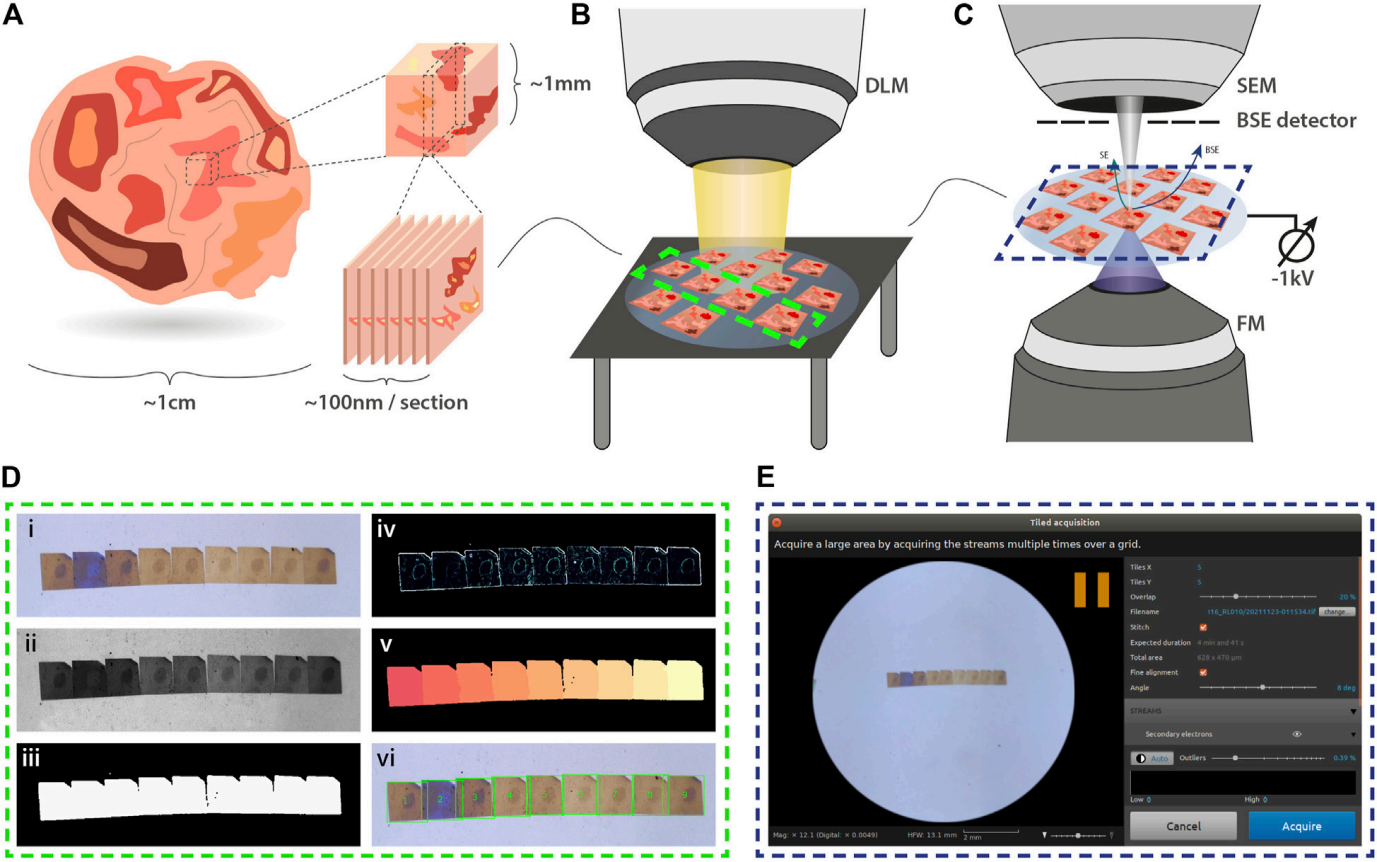
Integrated array tomography. **(A)** Ultrathin sections are prepared for simultaneous FM and EM imaging. **(B)** An overview image of the sections on the ITO-coated glass slide is then acquired by a digital light microscope (DLM). **(C)** Next, the sample is transferred to the integrated microscope for CLEM imaging. SEM imaging is performed using a −1kV bias potential applied to the sample stage to enhance BSE collection from the non-post-stained sections. **(D)** The overview image obtained by the DLM is used for instance segmentation of the serial sections. Descriptions of the image processing steps (i-vi) is provided in the main text. **(E)** The section overview image and bounding box coordinates of each serial section are fed to the microscope software to facilitate navigation.

Simple navigation between serial sections within the integrated microscope is crucial. Following joint EM-FM sample preparation (Figure 1A), the sections are imaged by a digital light microscope (DLM) (Figure 1B). To facilitate navigation within the integrated microscope (Figure 1C), the resulting overview image is used for detecting the boundaries of each section on the ITO-coated glass substrate via a segmentation routine^6^ (Figure 1D). The overview image (inset 1) is first contrast enhanced and converted to grayscale (inset 2). Intensity-based thresholding is used to create a binary mask image (inset 3), which is then applied to the grayscale image. To retrieve outlines of the section boundaries, the gradient is computed (inset 4). Watershed segmentation is then implemented by flooding the gradient image with a number of markers equal to the number of serial sections in the image (inset 5). The resulting labelled image (inset 6) then serves as input for navigation using a plugin within Odemis^7^, the open-source software that controls the microscope (Figure 1E).

### Targeted Correlative Acquisition of an Individual Region of Interest

**FIGURE 2:**
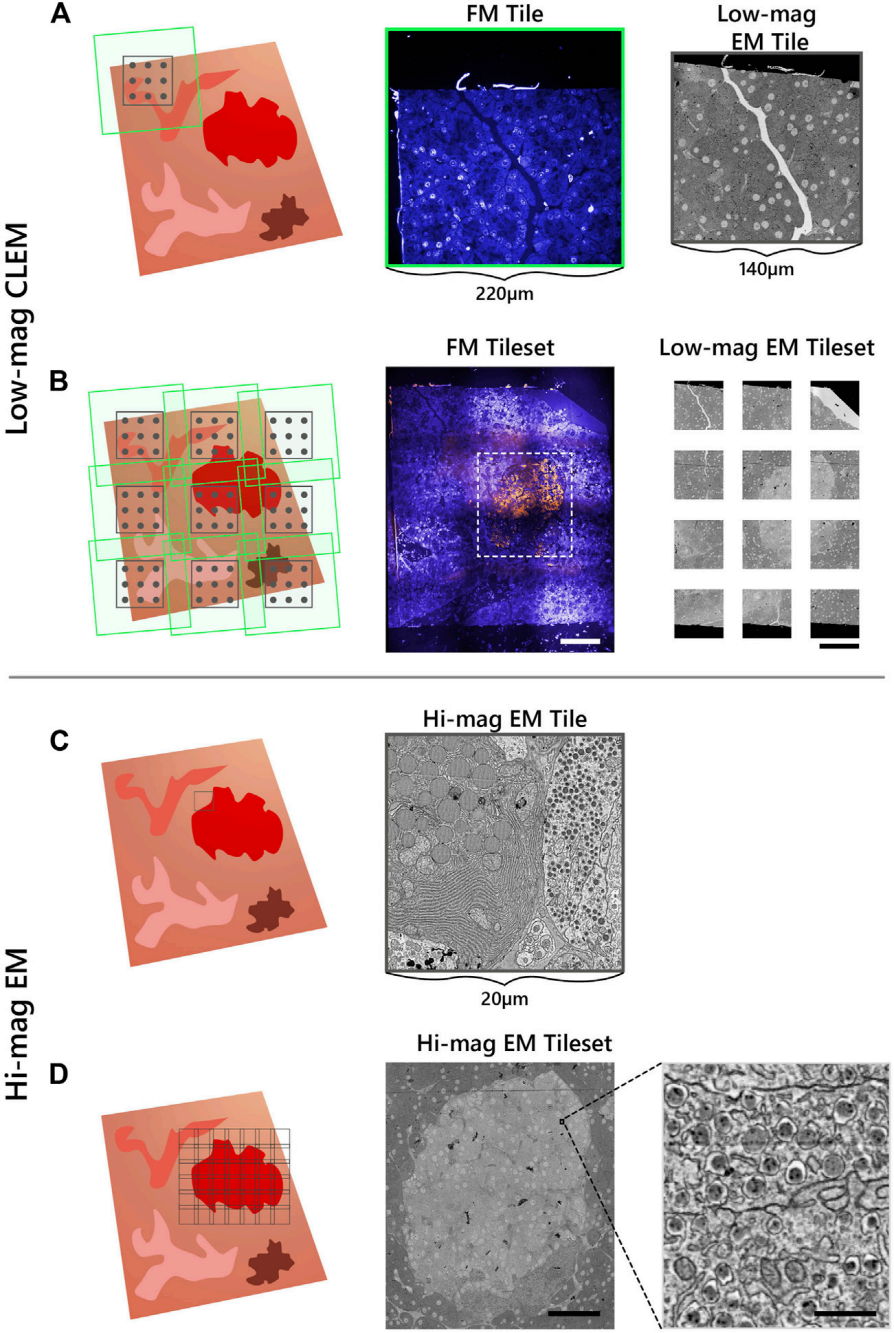
Integrated array tomography provides efficient, high-precision EM-FM imaging without bleaching of the fluorescence. **(A)** Acquisition of correlative FM (green outline) and low-magnification EM (black outline) images, followed by a registration procedure involving CL spots (grey circles) to register the image pair. **(B)** The stage is translated in a grid-like fashion such that there is sufficient overlap between neighboring FM image tiles—leaving a gap between adjacent EM tiles. **(C)** The fluorescence signal is used to identify targets for subsequent EM imaging (black outline). **(D)** The target ROI is captured by an automated tileset of high magnification EM tiles. Scalebars: **(B)** 100μm; **(D)** 50 μm (inset, 0.5 μm).

To identify ROI in the integrated microscope for subsequent EM acquisition, the correlative imaging scheme is engineered to obtain fluorescence overviews of each section, undamaged by the electron beam. The workflow starts by acquiring a FM and low-magnification EM image tile (Figure 2A). The FM tile is acquired prior to the EM tile to preserve the fluorescence signal. An automated registration routine guided by cathodoluminescent (CL) spots is then run to register the image pair (Haring et al., 2017). This sequence of correlative imaging is automatically repeated in a grid-like pattern, encompassing the entire section. FM image tiles are acquired with a 20% overlap such that they can be stitched together to allow for fluorescence-based ROI detection within each section (Figure 2B). The field width of the EM tile (∼140 μm) is chosen such that it spans the maximum extent possible without entering the overlap region of the neighboring FM image tiles,

$$w_{EM}=w_{FM}-2\;o_{FM}\;w_{FM}$$

where $w_{EM}$ and $w_{FM}$ are the respective EM and FM fields of view, and $o_{FM}$ is the overlap between adjacent FM tiles. In this way EM-FM registration is performed over as large an area as possible, while avoiding bleaching of the fluorescence. Fluorescence imaging of the entire section prior to EM would fulfil the same objective while circumventing the need for gaps between low-magnification EM image tiles. This would require manually registering the tilesets, however, as the transformation obtained from the CL registration procedure is unique to each image pair.

Fluorescence expression is then used to target areas for additional EM imaging at higher magnification (5 nm px^−1^) (Figure 2C). The ROI is manually navigated to via stage translation, whereby an automated tileset acquisition is initiated (Figure 2D). The tiles are spaced with a 10–15% overlap such that they can be stitched during post-processing. The correlative imaging pipeline is then repeated on the remaining serial sections.

### 2D Stitching and Correlation

**FIGURE 3:**
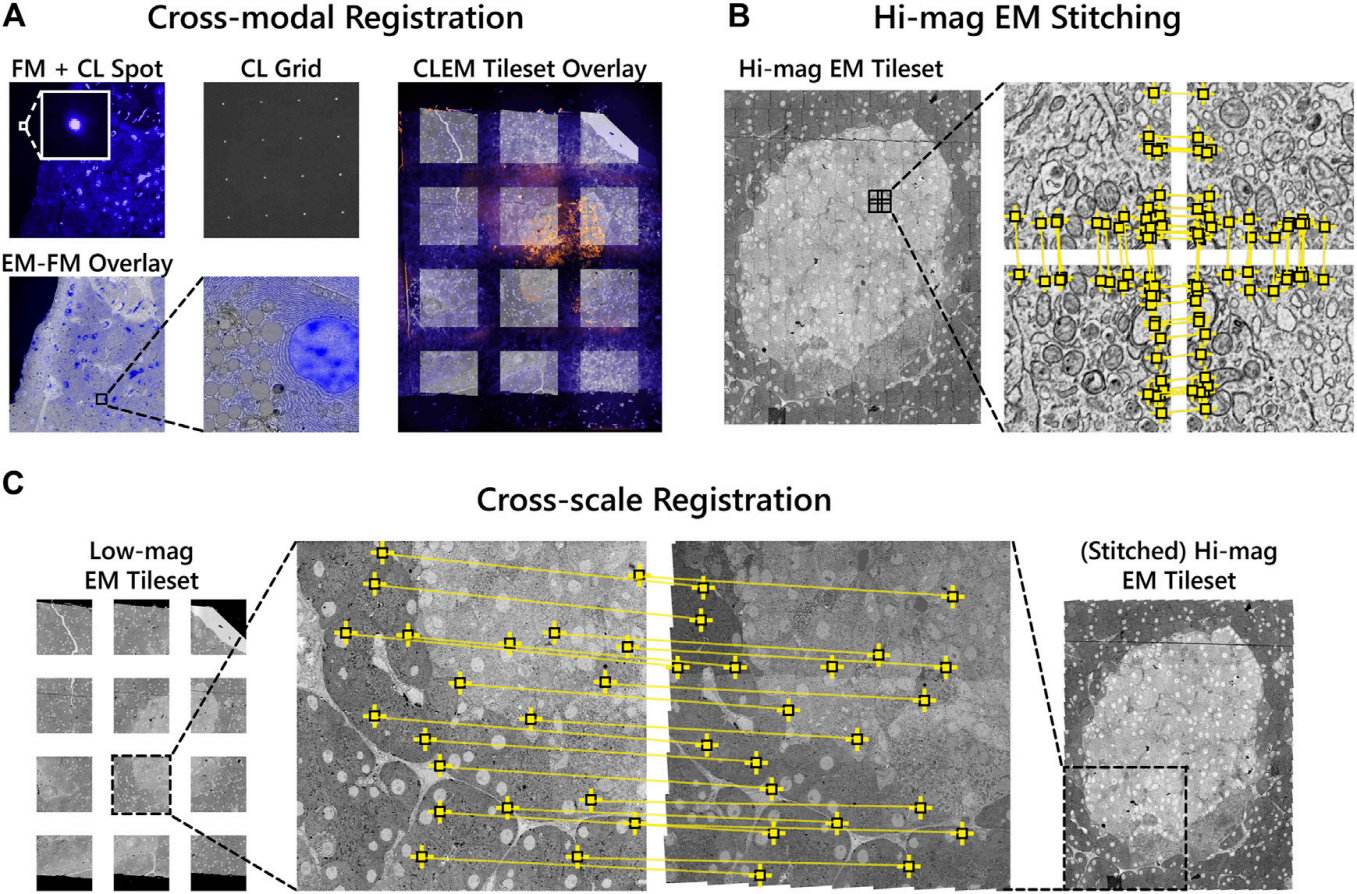
Correlative alignment routine registers tilesets across modalities and scales. **(A)** Automated registration procedure for registering FM and low-magnification EM image pairs using CL spots. FM tiles are then overlaid onto the low-magnification EM tiles of each section. **(B)** SIFT features (yellow squares) are extracted and used to stitch together neighboring high-magnification EM tiles within each section. **(C)** Low-magnification EM images are registered to the corresponding area of the stitched together high-magnification EM tileset. The low-magnification tiles thereby serve as a reference to ultimately overlay the fluorescence onto the high-magnification EM.

Overlaying the fluorescence onto the high-magnification EM requires correlating the datasets across different modalities and spatial scales. Each FM tile is first overlaid onto the corresponding low-magnification EM tile using the metadata generated by the CL registration procedure. A grid of CL spots is recorded with the camera of the fluorescence microscope in the absence of excitation light (Figure 3A). The appropriate affine transformation is calculated by localizing each CL spot and matching it with the known position of the electron beam (“cross-modal” registration) (Haring et al., 2017). The stage coordinates are extracted to then correlate and position each image pair in the tileset (Figure 3A).

The high-magnification EM tileset is stitched independently of both the FM and low-magnification EM tiles (Figure 3B). Stage coordinates are used to first establish a set of potential neighboring tiles. For each tile, SIFT features are extracted and matched between the candidate neighbors. Affine transformation parameters for each tile are then estimated by minimizing the squared distance between corresponding features (Saalfeld et al., 2012; Khairy et al., 2018).

Next, the low-magnification image tiles are registered to the corresponding area of the stitched high-magnification EM tileset (“cross-spatial” registration, Figure 3C). Stage coordinates are used to determine the set of high-magnification tiles that overlap with each low-magnification tile. A composite image of the overlapping tiles is rendered, processed with SIFT, and matched with the features in the low-magnification tile. The affine transformation computed from the feature matching is then propagated to each of the FM tiles such that they are overlaid onto the high-magnification EM tileset. In this way, the low-magnification EM serves as a proxy to correlate the fluorescence to the high-magnification EM. The overlay accuracy is reduced in the areas between low-magnification tiles where the transformation is extrapolated (Supplementary Figure S1A—F). This can be corrected for via (manual) landmark registration by e.g. aligning the Hoechst signal to nuclei recognized in the EM using software such as ec-CLEM (Paul-Gilloteaux et al., 2017), which is routinely used for image registration in sequential CLEM experiments (Franke et al., 2019; Tuijtel et al., 2019; Lee et al., 2020). In general, the overlay accuracy cannot be expected to be below the pixel size of the low magnification EM.

### Correlative 3D Reconstruction

**FIGURE 4:**
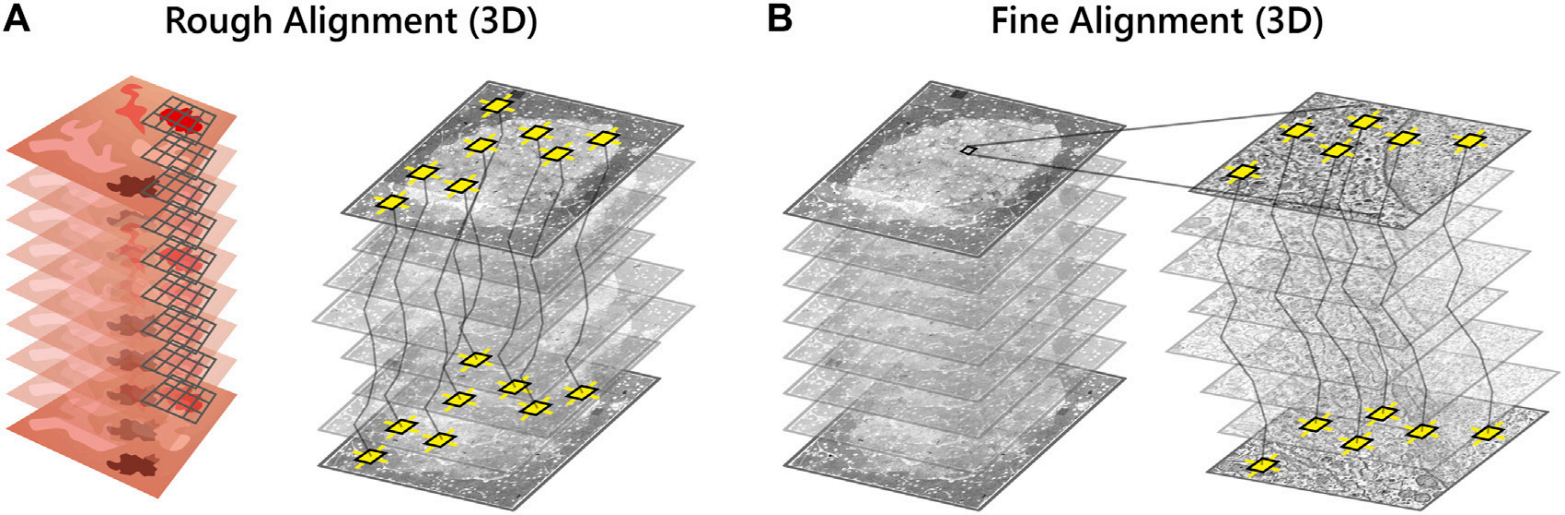
Volume reconstruction of the high-magnification EM stack. **(A)** SIFT features (yellow squares) are used to roughly align the high-magnification EM stack in *z*. A downsampled image of each section is rendered as the full resolution EM tileset is too large (several GB) for feature extraction. **(B)** The EM stack alignment is refined by least squares optimization of the displacement between matched features.

A robust and scalable solution is required for volume alignment of the high-magnification EM stack, the “backbone” of the multimodal dataset. The stitched sections are downsampled and roughly aligned in z (Figure 4A) to facilitate feature mapping between image tiles in adjacent sections. A system of linear equations consisting of SIFT features is then solved to finely align the image stack in 3D (Figure 4B) (Khairy et al., 2018). The features extracted during stitching are reused, enabling a faster and more efficient reconstruction of the EM volume.

**FIGURE 5:**
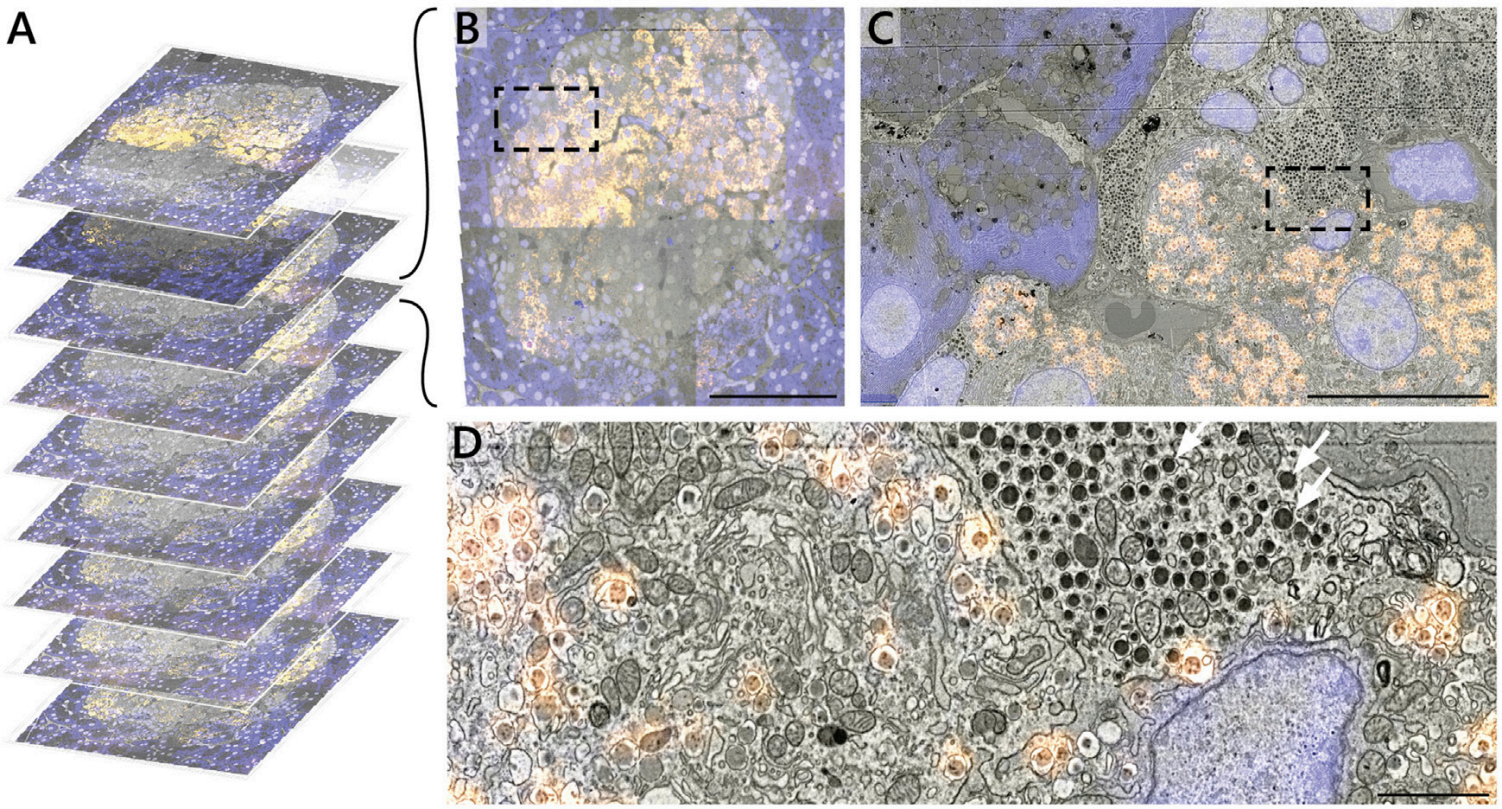
Correlative reconstruction of nine sections of pancreas. **(A)** 2D and 3D alignment routines are combined to yield a CLEM stack of nine serial sections of rat pancreas tissue. **(B–D)** CLEM imaging of the islet of Langerhans at varying spatial resolution. Hoechst (blue) and AF594 (orange) fluorescence signals are superimposed onto the EM ultrastructure. The AF594 signal, in particular, facilitates recognition of insulin granules from e.g. non-fluorescent glucagon granules (white arrows, D). Scale bars: **(B)** 50μm; **(C)** 5μm; **(D)** 1 μm. Raw data at full resolution is available via Nanotomy.

The 2D correlative alignment procedure (Figure 3) is then run on each section, mapping the fluorescence onto the high-magnification EM volume. The nine serial sections of rat pancreas were thereby used to realize a proof-of-concept of the iCAT workflow (Figure 5A). An islet of Langerhans was identified from anti-insulin immunofluorescence of AF594 and chosen for subsequent, high-magnification EM imaging (Figure 5B). The fluorescence data clearly delineates the endocrine region from the surrounding exocrine tissue, which is characterized by dense endoplasmic reticulum (ER) and the absence of insulin labeling (Figure 5C). Although it was chosen as a nuclear marker, Hoechst also binds to the RNA present in the ER. The endocrine region, in contrast, is characterized by an abundance of insulin-secreting beta cells with distinct nuclei. The high EM-FM registration accuracy afforded by iCAT enables a clear distinction between different types of granules present in the endocrine tissue (Figure 5D). Discerning insulin from other hormone granules is nontrivial as all are roughly 100 nm in diameter. Making this differentiation from EM data alone requires expert-level interpretation.

By limiting high-magnification EM to only the islet, the total imaging volume is reduced by a factor ∼10 with respect to the full section volume (0.03 mm^2^ per islet vs 0.4 mm^2^ per section). Similar reductions are realized in the total dataset size (0.1 vs ∼1 TB) easing data management requirements. This initial proof of concept was designed around only a limited number of serial sections to more efficiently optimize each procedure in the workflow.

### Proof of Concept on Zebrafish Pancreas Tissue

**FIGURE 6:**
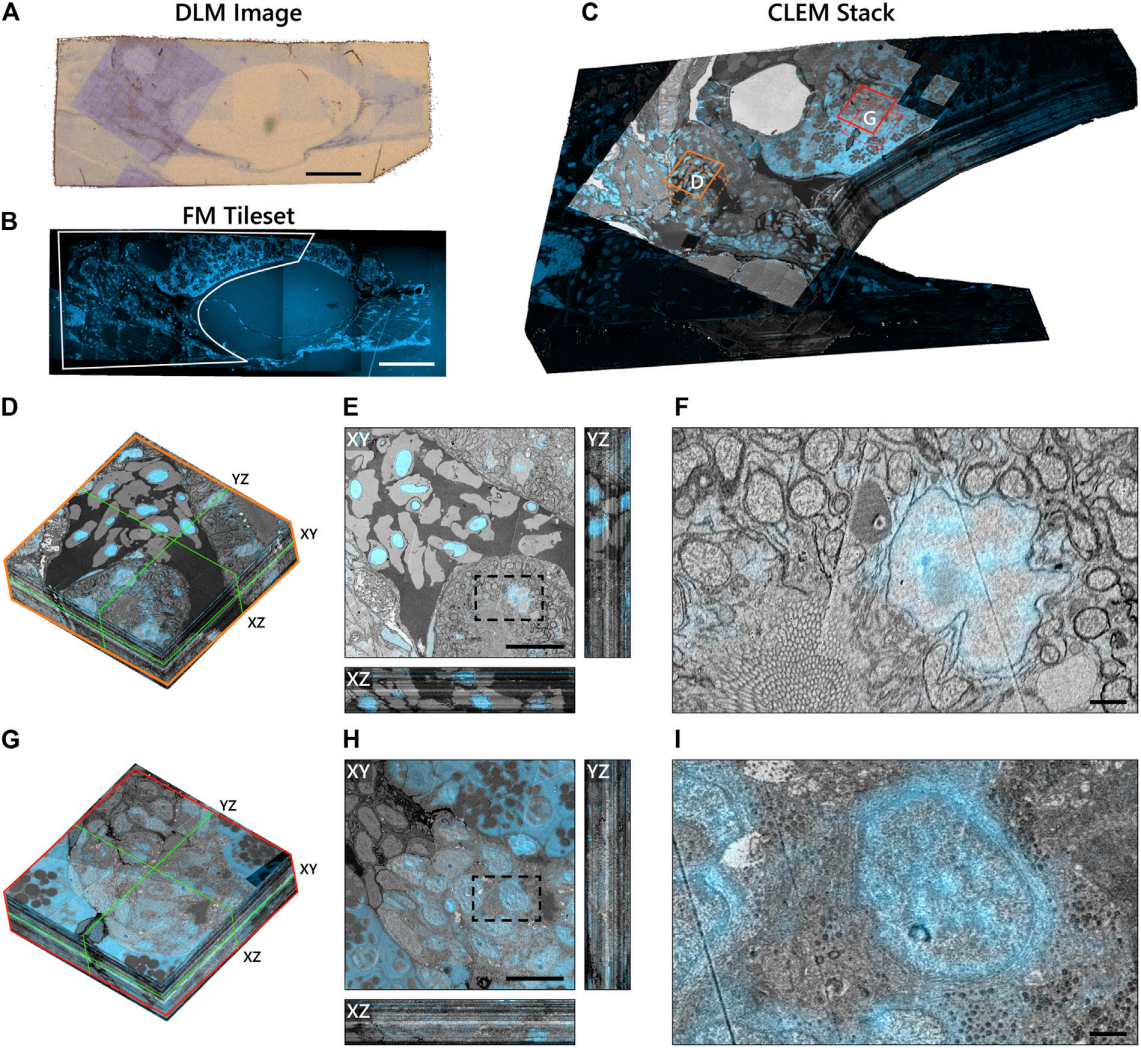
Integrated correlative array tomography applied to 63 serial sections of zebrafish pancreas. **(A)** Contrast-enhanced optical image of an individual serial section obtained by the DLM. The section was re-acquired post-EM imaging, revealing the region of interest irradiated by the electron beam. The biological material can be seen in pale blue in contrast with the bare EPON (brown background). **(B)** Hoechst signal of an individual serial section, outlining (white) the portion of the tissue shown in **(C)**. **(C)** CLEM volume of the zebrafish tissue cropped to the ROI selected for high-magnification EM imaging—plus a portion of the surrounding fluorescence signal. Sub-stacks for inspection are denoted by orange **(D–F)** and red **(G–I)** boxes. **(D)** 3D sub-stack of muscle tissue within the zebrafish pancreas. Green lines indicate orthoslices of the XY, XZ, and YZ planes shown in **(E)**. **(F)** Zoomed-in region of the XY plane showing high-precision FM overlay of Hoechst onto a cell nucleus. **(G–I)** Same as in **(D–F)**, but for an islet of Langerhans. Ill-defined cell and organelle membranes in **(H)** and **(I)** indicate suboptimal preservation of the ultrastructure in this region of the pancreas. Scale bars: **(A,B)** 100μm; **(E,H)** 10μm; **(F,I)** 1 μm. Raw data at full resolution is available via Nanotomy.

To demonstrate the scalability of the workflow, we applied it to a larger volume of larval zebrafish (Figure 6). The Hoechst signal was useful in identifying the exocrine region of the pancreas (Figure 6B) as the insulin immunofluorescence from TRITC was weak. TRITC was chosen for its stronger fluorescence in vacuum compared to Alexa dyes (manuscript in preparation); potential causes for the weak immunofluorescence in the zebrafish pancreas are still under investigation. The exocrine region, encompassing an islet of Langerhans, together with the underlying muscle tissue was selected for high magnification EM. The reconstruction of the CLEM volume was cropped to remove the background fluorescence in the swim bladder (Figure 6C). Sub-stacks within the correlative volume were then extracted for further analysis (Figures 6D,G). Note that the ultrastructure is better preserved in most tissues than in the islet (Figures 6H,I), a phenomenon previously seen in other species (unpublished results).

We generally observe high EM-FM overlay precision as evidenced by the Hoechst signal confined to the nuclear envelope in the muscle tissue (Figures 6E,F). The registration accuracy does, however, exhibit variability in several sections (Movies A.1 and A.2)—more so than is seen in the rat pancreas tissue where it appears limited to the gaps between low-magnification EM tiles. In these instances, the inaccuracy stems from a malfunction in the CL registration procedure itself (Supplementary Figure S1G—N). Variations in the EM image intensity, particularly in the islet, can also be observed for a number of sections (Figure 6H: *XZ* and *YZ* cross-sections). We attribute these artifacts primarily to ultrastructure preservation as they do not appear to be as prevalent in the muscle tissue. While the SEM imaging parameters and detection settings were held constant throughout the acquisition, day-to-day changes in the environment (e.g. temperature, humidity levels) may have varied.

In total, 66 sections were prepared, of which three (*z* = 9, 10, 34) were discarded due to excess surface debris. The omission of consecutive sections was mitigated by extending the SIFT feature depth search from 2 to 3 such that sections *z* = 8 and *z* = 11 could be registered. Total acquisition times for low-magnification CLEM and high-magnification EM were 7.2 and 71 h respectively, versus 335 h for full section imaging at high-magnification.

## Discussion

A new workflow for integrated array tomography for the semi-automated acquisition and reconstruction of volume CLEM data is presented. High-resolution EM is limited to select ROI by targeting areas based on fluorescence expression. This not only expedites acquisition time, but eases the burden on data management requirements. Interpretation of EM data is in turn facilitated by the addition of fluorescent labels. The workflow demonstrated here extends the work of Liv et al. (Liv et al., 2013), which introduced the integrated microscope, and Haring et al. (Haring et al., 2017), which presented the fiducial-free CL registration procedure, to targeted correlative imaging of serial sections. Gabarre et al. (Gabarre et al., 2021) presented an alternative method for integrated array tomography in which light microscopy and EM are combined to localize structures through a series of feedback loops. Our approach differs in several ways. First, fluorescence imaging is done *in-vacuo* as opposed to transmitted light microscopy done at ambient pressure. This allows for more automated EM-FM (or EM-LM) overlay, as the CL registration procedure can only be done in high vacuum (Haring et al., 2017). Additionally, the multi-modal alignment methodology conceived here offers a more scalable solution for generating volumetric CLEM data. Integrated array tomography was inspired in part by the multi-scale approach of Hildebrand et al. (Hildebrand et al., 2017), in which full brain EM imaging of a larval zebrafish was conducted by selecting ROI for subsequent acquisition based on inspection between imaging rounds. In this work, conversely, ROI are identified by *in-situ* fluorescence, bypassing the need for post-processing and alignment between magnification scales.

On-section immunofluorescence and fluorescent staining constitute viable options for FM imaging of resin-embedded sections in high vacuum. Pancreas tissue in particular is well-suited for immunofluorescence due to the prevalence of insulin epitopes. While in both nature and technique development, immunolabeling approaches are always dependent on the capacity for antibodies and epitopes to interact, this is typically inefficient for most antibodies, and particularly so for EPON-embedded sections. We find that approximately 1 in 10 antibodies tested in our lab are applicable for EPON labeling. While acrylic resins (e.g. Lowicryl, LR White) have been shown to be more compatible with immunolabeling, a trade-off must be made between the strength of the fluorescence signal and the quality of the ultrastructure (Watanabe et al., 2011; Paez-Segala et al., 2015). Complications with serial sectioning and ultrastructure preservation (beyond that shown in the zebrafish pancreas) arose when experimenting with Lowicryl; hence EPON was selected as the embedding medium for this study.

Probes typically used for live FM, such as fluorescent proteins, are likewise incompatible with conventional EM sample preparation techniques (de Boer et al., 2015). Although protocols have been developed for retaining fluorescence post-embedding (Kukulski et al., 2011; Watanabe et al., 2011; Peddie et al., 2014; Fu et al., 2020), the same compromises exist between fluorescence retention and ultrastructure preservation. Fluorescent proteins have the additional limitation that the specimen must be genetically modified, rendering them unsuitable for use in native animals and humans. In-resin fluorescence preservation thus remains a challenge—only made more difficult by imposing high vacuum conditions, which may lower fluorescence intensities for biological probes typically optimized for use in aqueous environments (Peddie et al., 2014). We are nevertheless confident that future developments in fluorescent proteins and embedding media will present compelling opportunities to apply integrated array tomography to a variety of biological questions.

We foresee that the multimodal datasets obtained using this method will be instrumental in forthcoming machine learning applications (Eckstein et al., 2020; Liu et al., 2020; Heinrich et al., 2021). Thus far, applications of registered EM-FM datasets appear to be limited to facilitating registration of sequential CLEM data using artificial predictions for the fluorescence signal (Ounkomol et al., 2018; Seifert et al., 2020). Volume EM datasets, particularly in connectomics, are now routinely segmented via deep convolutional neural networks (Buhmann et al., 2021; Heinrich et al., 2021). Acquisition rates and manual annotation of datasets, however, both serve as bottlenecks for reconstructing dense networks of cells and organelles (Kornfeld and Denk, 2018). Given its ability to provide labeled biological information as well as reduce imaging volumes to select regions, integrated array tomography is poised to deliver significant gains in this arena.

Future work will be directed towards further refinement and automation. The CL registration procedure could be made more robust by illuminating the sample with a greater number of CL spots or by increasing the camera integration time. Updates to the alignment software could furthermore allow for the distortion field correction used in Haring et al. (Haring et al., 2017) to achieve sub-5nm overlay precision. Cutting sections manually remains a significant bottleneck for throughput, as it is prone to error and requires expert training (Wanner et al., 2015). We expanded from a single section to nine, to 63, and have now placed more than 100 serial sections onto ITO-coated coverslips. Increasing beyond ∼10 μm of biological material, however, is cumbersome without more sophisticated sectioning techniques such as automated tape-collecting ultramicrotome (ATUM) (Hayworth et al., 2014) or magnetic collection (Templier, 2019). These may introduce their respective complications; ATUM, for example, is designed to collect sections on (opaque) Kapton tape. More extensive automation strategies can alternatively be applied to the correlative imaging pipeline. Delpiano et al. (Delpiano et al., 2018) devised a way to automatically detect fluorescent cells using an integrated light and electron microscope. We envision a workflow for fully automated integrated array tomography in which fluorescent ROIs are automatically recognized, navigated to, and acquired, rendering three-dimensional CLEM datasets tailored to answer the specific biological research question.

## Data Availability

The datasets presented in this study are available from the Nanotomy repository (http://www.nanotomy.org/OA/Lane2022FiCD/).

## Footnotes

1
https://github.com/saalfeldlab/render.

2
https://github.com/hoogenboom-group/iCAT-workflow.

3
https://github.com/AllenInstitute/render-python.

4
https://github.com/AllenInstitute/BigFeta.

5
https://imagej.net/plugins/volume-viewer.

6
https://github.com/hoogenboom-group/secdetect.

7
https://github.com/delmic/odemis.

## Supplementary Material

Supplementary Figure S1 Reduced overlay accuracy due to extrapolation and errors in the CL registration procedure. Overlay (in)accuracy is a combination of the errors in the cross-modal and cross-spatial registration procedures. **(A)** Partial low-magnification CLEM tileset of rat pancreas from which features inside **(B-D)** and outside **(E-F)** a low-magnification EM tile were selected to assess the overlay accuracy (denoted by white squares). **(B)** Cross-modal registration error for a cell nucleus in low-magnification CLEM, measured by calculating the centroid of the nucleus in both modalities and computing the relative displacement. **(C)** Cross-spatial registration error as measured by the phase correlation between the low and high-magnification EM. **(D)** Sub-100nm overlay accuracy for the cell nucleus in high-magnification CLEM. **(E-F)** Same as in **(B)** and **(D)** for a cell nucleus acquired outside of a low-magnification EM tile. Overlay accuracy is reduced to several hundred nanometers due to the imprecision incurred by extrapolating the cross-modal registration. **(G)** Partial low-magnification CLEM tileset of zebrafish pancreas from a section in which the CL registration procedure achieved the expected precision. **(H-J)** Same as in **(B-D)** but for a nucleus in the zebrafish pancreas. **(K-N)** Same as in **(G-J)** but for the section with the lowest apparent overlay accuracy (1.5μm). The inaccuracy is dominated by an error in the cross-modal registration as the phase correlation **(M)** shows a sub-50nm translation. Possible causes for the error include poor CL spot localization due to noise in the CL signal. Scale bars: **(A)** 50μm; **(B, D, E, F)** 0.5μm; **(G, K)** 50μm; **(H, J, L, N)** 1μm.
