# Supplementary figures and images for: Integrated Array Tomography for 3D Correlative Light and Electron Microscopy
Source: Front Mol Biosci. 2022 Jan 19;8:822232. doi: 10.3389/fmolb.2021.822232 (PMC8809480; doi:10.3389/fmolb.2021.822232)

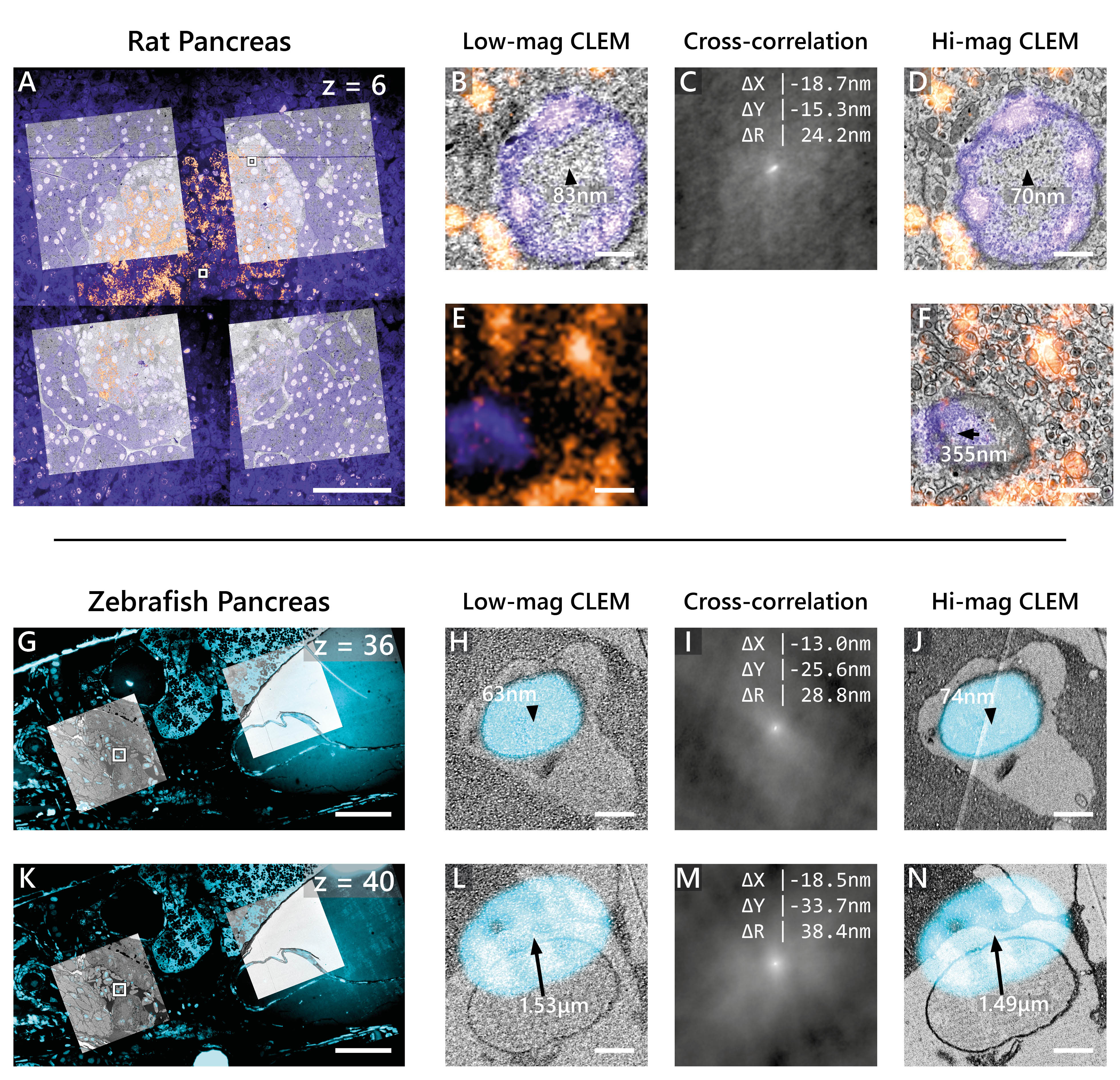

Supplement: Supplementary file 1 [file Image1.jpeg]
